# Pharmacological Intervention to Modulate HDL: What Do We Target?

**DOI:** 10.3389/fphar.2017.00989

**Published:** 2018-01-22

**Authors:** Nicholas J. Woudberg, Sarah Pedretti, Sandrine Lecour, Rainer Schulz, Nicolas Vuilleumier, Richard W. James, Miguel A. Frias

**Affiliations:** ^1^Hatter Institute for Cardiovascular Research in Africa and South African Medical Research Council Inter-University Cape Heart Group, Department of Medicine, Faculty of Health Sciences, University of Cape Town, Cape Town, South Africa; ^2^Division of Endocrinology, Diabetes, Hypertension and Nutrition, Department of Internal Medicine Specialities, Faculty of Medicine, University of Geneva, Geneva, Switzerland; ^3^Institute of Physiology, Justus Liebig University Giessen, Giessen, Germany; ^4^Division of Laboratory Medicine, Department of Genetics and Laboratory Medicine, Geneva University Hospitals, Geneva, Switzerland

**Keywords:** HDL, pharmaceutical intervention, HDL functionality, HDL subclass, cardiovascular disease

## Abstract

The cholesterol concentrations of low-density lipoprotein (LDL) and high-density lipoprotein (HDL) have traditionally served as risk factors for cardiovascular disease. As such, novel therapeutic interventions aiming to raise HDL cholesterol have been tested in the clinical setting. However, most trials led to a significant increase in HDL cholesterol with no improvement in cardiovascular events. The complexity of the HDL particle, which exerts multiple physiological functions and is comprised of a number of subclasses, has raised the question as to whether there should be more focus on HDL subclass and function rather than cholesterol quantity. We review current data regarding HDL subclasses and subclass-specific functionality and highlight how current lipid modifying drugs such as statins, cholesteryl ester transfer protein inhibitors, fibrates and niacin often increase cholesterol concentrations of specific HDL subclasses. In addition this review sets out arguments suggesting that the HDL3 subclass may provide better protective effects than HDL2.

## Introduction

Cardiovascular disease (CVD) are the leading cause of death globally (GBD 2013 Mortality and Causes of Death Collaborators, 2015). It is predicted that the number of deaths from CVD will increase to 23.3 million by 2030 (Mathers and Loncar, 2006). Blood lipids have traditionally served as accurate risk factors for cardiovascular events. Increases in low-density lipoprotein (LDL) cholesterol and decreases in high-density lipoprotein (HDL) cholesterol rise cardiovascular risk (Gordon et al., 1977; Barter and Rye, 1996; Jeppesen et al., 2000; Kontush et al., 2003). The protective effect of HDL has been primarily attributed to reverse cholesterol transport (RCT). This process removes cholesterol from macrophages and other cells residing in the blood vessel wall and exports it to the liver, thus reducing LDL contribution to the development of atherosclerotic plaques and therefore reducing the risk of an ischemic event (Barter and Rye, 1996; Yu X.H. et al., 2013). HDL had long been considered as a primary therapeutic target for lowering the risk of atherosclerotic disease. Despite the development of effective HDL-raising drugs, large-scale clinical trials showed disappointing results with no significant reduction of clinical cardiovascular events (Boden et al., 2011; Schwartz et al., 2012). Whilst this may indicate that targeting an increase in HDL cholesterol (HDL-C) should be reconsidered we argue that this, in fact, underlines not only the complexity of HDL particle metabolism but also the complexity of HDL structure, composition and function. It also suggests that focusing on improving HDL quality rather than just increasing HDL quantity may be the next target for future therapies. Historically, HDL particles have been subdivided in several subclasses according to their density, but these subdivisions also reflect differences in their functional properties as defined by the different proteins and lipids associated with the subclasses.

The aim of this review is to consider the different HDL subclasses as one possible target for more efficient cardiac protection. The different HDL subclasses will be described and evidence regarding the superiority of particular HDL subclasses, undoubtedly reflecting their composition for improved cardioprotection will be discussed. Finally, this review will attempt to explain the failures observed in past and current HDL therapies for CVD.

## HDL: Structure, Composition, and Function

There are presently two major, interlinked objectives of research into HDL. One is to understand the mechanisms by which HDL protects against CVD (functionality), and how these mechanisms are compromised in different pathological states. The second objective is clinical and aims to identify HDL parameters that more accurately estimate cardiovascular risk as well as providing diagnostic tools applicable in the clinical laboratory. The following section will review various characteristics of HDL with these two objectives in mind.

## Definition

**FIGURE 1:**
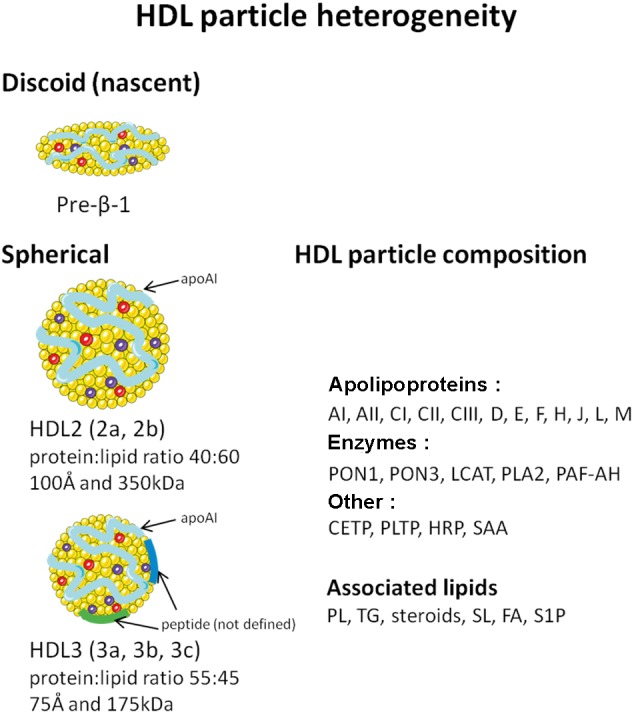
Schematic representation of HDL heterogeneity. PON, paraoxonase; LCAT, lecithin-cholesterol acyltransferase; PLA2, phospholipase A2; PAF-AH, platelet activating factor acetylhydrolase; CETP, cholesterol ester transfer protein; PLTP, phospholipid transfer protein; HRP, haptoglobin related protein; SAA, serum amyloid AI and AII; PLs, phospholipids; TG, triglyceride; SLs, sphingolipids; FA, fatty acid; S1P, sphingosine-1-phosphate.

Of the major lipoprotein classes, HDL are defined by their high protein:lipid ratio and the predominant presence of apolipoprotein (apo) AI, which accounts for approximately 70% of the total protein content of the lipoprotein and 30–40% of total protein–lipid content (Gillard et al., 2009). ApoAII is the other major HDL apolipoprotein, accounting for 10–15% of total protein content. HDL are the most heterogeneous of the lipoproteins, varying in buoyant density, electrophoretic mobility, size, protein and lipid composition (**Figure 1**). More than 80 proteins and 150 lipids have been shown to be associated with HDL particle. Thus, it is highly unlikely that each lipoprotein particle carries the same complement of protein or lipid components. Reflecting, in part, metabolic processing within the plasma compartment. Lipid poor apoAI (of hepatic or intestinal origin) acquires increasing quantities of phospholipids and cholesterol, maturing through nascent discoidal HDL (preβ-1 HDL) to form spherical HDL (**Figure 1**) (Rye and Barter, 2014). The latter arises from esterification of acquired cholesterol by lecithin-cholesterol acyltransferase (LCAT), and absorption of triglycerides that create a hydrophobic core, which must be shielded from the aqueous environment by amphipathic phospholipids and proteins. In contrast to other plasma lipoproteins, where the whole particle is eliminated, the cholesterol component alone of spherical HDL is transferred to the hepatocyte. The residual, lipid poor apoAI becomes available to recycle through the maturation process, before eventual renal excretion (Rye and Barter, 2014). These factors add several levels of complexity to attempts either to correlate serum HDL with cardiovascular risk, or define the functions of the lipoprotein.

In terms of clinical relevance, it is presently the cholesterol component of HDL that is of primary importance. A wide range of retrospective and prospective epidemiological studies have consistently demonstrated its inverse correlation with the incidence of atherosclerotic disease (Gordon et al., 1977; Assmann et al., 1996; Barter and Rye, 1996; Goldbourt et al., 1997). It is thus still incorporated in clinical guidelines as one of the primary parameters for assessing cardiovascular risk (Piepoli et al., 2016). Perhaps surprisingly, cholesterol is a relatively minor component of HDL. It represents 15% by weight. Unfortunately, recent clinical trials that attempted to reduce cardiovascular risk by pharmacologically increasing the cholesterol content of HDL have been unsuccessful (Kühnast et al., 2015). This has provoked a re-think of the mechanisms by which HDL may protect the vascular system, and given greater weight to the heterogeneity of HDL subtypes as a factor in the multiple functions of the lipoprotein.

## HDL Fractionation and Isolation

Several of the properties of HDL outlined above have been exploited to fractionate HDL subclasses. These include HDL electrophoretic mobility which utilizes non-denaturing, two-dimensional gel electrophoresis to fractionate of HDL revealing the presence of the subclasses (Asztalos et al., 2011). These can be visualized and quantified by appropriate staining techniques. However, the procedure does not lend itself to isolation of HDL subclasses.

HDL buoyant density techniques exploit the protein/lipid ratio of lipoproteins to allow sequential lipoprotein flotation by ultracentrifugation in a high salt medium (Lindgren et al., 1972). The major lipoprotein classes can be separated due to their differing protein:lipid ratios and types of lipid. Ultracentrifugation is the principal procedure used to isolate HDL subfractions, predominantly HDL2 and HDL3. These can be further divided by more sophisticated centrifugation procedures into subclasses HDL2a and b and HDL3a, b, c, as illustrated in **Figure 1** (Rosenson et al., 2011).

Whilst numerous proteins are associated with HDL, their concentrations are minor compared to that of apoAI. Alaupovic (2003) suggested that such protein heterogeneity could be exploited to define discrete HDL subclasses. As the proteins define HDL function, isolation via these components could give rise to subtypes of greater functional homogeneity. The procedure requires monospecific antibodies that can be used to prepare affinity absorption columns. One major drawback is that the acidic elution conditions can adversely affect the function of the isolated subclasses.

## HDL Subclass Structure and Function

### Preβ-1 HDL

These are structurally the simplest form of HDL. They consist of 1–2 molecules of apoAI with a layer of phospholipid molecules and trace amounts of cholesterol. Preβ-1 HDL accounts for only 5–6% of plasma HDL, in part because their high capacity to absorb phospholipids and cholesterol rapidly converts them to other HDL species. Preβ-1 HDL are hypothesized to be the first link in the chain of events preventing the development of atherosclerotic plaque, because of their function is to avidly remove cholesterol (the non-esterified form) and phospholipids from cells via binding to the cell surface, ATP binding cassette A1 and G1 (ABCA1 and ABCG1) transporter protein. Studies supporting this function have established that the plasma content of preβ-1 is a major determinant of the rates of cholesterol efflux from macrophages (de la Llera-Moya et al., 2010).

### HDL Subclasses 2 and 3

Spherical HDL is a spectrum of lipoprotein particles that covers the density range *d* 1.063–1.21 g/ml. HDL3 occupies the density range *d* 1.125–1.21 g/ml. It is protein enriched (mean protein:lipid ratio 55:45 by weight), with a mean particle diameter of 75 Å and a mean molecular weight of 175 kDa. HDL2 occupies the lower density range of the spectrum (*d* 1.063–1.125 g/ml), reflecting lipid enrichment (protein:lipid ratio 40:60) compared to HDL3. Its mean diameter and molecular weight (100 Å and 350 kDa, respectively) are correspondingly greater than HDL3 (Chapman, 1986) (**Figure 1**). HDL3 is the predominant HDL subclass. Women have significantly higher concentrations of HDL, both for HDL3 (∼25%) and notably for HDL2 (two to threefold higher). ApoAI and AII are major structural peptides of both subclasses. However, HDL3 is enriched in apoAII compared to HDL2 (a fivefold lower apoAI:AII concentration ratio than in HDL3).

With respect to the major lipid components of the subclasses (esterified and free cholesterol, phospholipids, triglycerides) there is no marked difference in concentrations between HDL2 and HDL3. Nevertheless, the relative concentrations (% total mass) of esterified cholesterol and phospholipids are greater in the HDL2 subclass, reflecting increased lipid content. However, increased HDL3 concentration implies that with respect to serum concentrations, HDL-associated cholesterol is present in greater concentrations in HDL3, notably for men.

### HDL Subclass Definition According to Protein Content

Proteomics has allowed for identification of differences in the protein profiles of HDL2 and HDL3 (Davidson et al., 2009). As noted above, apoAII is more present in HDL3 compared to HDL2. A second feature, which may impact on function, is the enrichment of minor proteins in the HDL3 subclass compared to HDL2 (**Table 1**). Such studies are also revealing clusters of proteins within HDL that have common functions linked to particular activities, including complement activation, the innate immune response, oxidative stress and regulation of proteinase activity, through which a number of pathological processes could be influenced (Vaisar et al., 2007; Davidson et al., 2009). For example, paraoxonase (PON)-associated HDL are associated with the anti-coagulant protein S (Moren et al., 2016) and transthyretin (TTR) or prealbumin can be differentially associated in HDL in patients with differing risk of CVD (Cubedo et al., 2012).

**Table 1:** Relative distribution of peptides between HDL2 and HDL3.

Preferentially in HDL3	Preferentially in HDL2
Paraoxonase-1 (PON1)	Apolipoprotein CI
Paraoxonase-3 (PON3)	Apolipoprotein CII
Apolipoprotein F	Apolipoprotein CIII
Apolipoprotein L-I	Apolipoprotein E
Apolipoprotein J (clusterin)	
Apolipoprotein M	
Apolipoprotein D	
Apolipoprotein A-IV	
PAF-acetylhydrolase	
Serum amyloid AI and AII	
Haptoglobin related protein	

### HDL and Lipid Content

In a recent study, Serna et al. (2015) identified more than 150 different lipids in HDL particles. Quantitatively, phospholipids (phosphatidylcholine and sphingomyelin) are the main constituents of the HDL lipidome (40–60%), followed by esterified cholesterol (30–40%), triglycerides (5–12%), and free cholesterol (5–10%) (Wiesner et al., 2009). Changes in lipid composition can occur and may alter the atheroprotective capacities of HDL (Salazar et al., 2015). Little work has been undertaken on the distribution of the minor lipid components between the subclasses. It has been reported that in general sphingolipids are less enriched in HDL3, which, together with a lower free cholesterol content (also present in the outer lipid layer of lipoproteins) may impact on surface lipid fluidity and thus lipoprotein function (Kontush et al., 2013). In contrast, sphingosine-1-phosphate (S1P), a particularly bioactive lipid, is preferentially associated with HDL3 (Kontush et al., 2007; Serban et al., 2014). S1P is generated intracellularly from sphingomyelin and is transported to extracellular environment where it binds to HDL (Mitra et al., 2006). The diverse atheroprotective functions of HDL and the mechanisms by which these effects are achieved have been in many cases linked to the S1P content of HDL. These include, preventing ischemic injury (Theilmeier et al., 2006; Frias et al., 2012); reducing cytotoxicity (Kimura et al., 2001; Kontush et al., 2007); inducing prostacyclin release (Liu et al., 2012) and preventing LDL oxidation (Kontush et al., 2007; Rodríguez et al., 2009). In patients with coronary artery disease (CAD), the content of S1P in HDL particle was lower, and could be raised using *in vitro* S1P loading (Sattler et al., 2010, 2015). This observation was recently extended to patients with coronary in stent restenosis (Jing et al., 2015) and in type 2 diabetic patients (Brinck et al., 2016). In this very recent paper we showed that the content of S1P is inversely correlated with glycated hemoglobin (HbA1c) in type 2 diabetic patients and the concentration of S1P is directly correlated with its cardiac specific anti-apoptotic capacity (Brinck et al., 2016).

Research is now centered on understanding the different activities associated with HDL, how they impact on cardiovascular physiology and pathophysiology beyond lipid transport and how they may contribute to the global cardioprotective effect of lipoprotein. The highly heterogeneous nature of HDL, reflecting the complex metabolic process to which it is subjected in serum, whilst complicating attempts to characterize the lipoprotein, may also provide a framework for compartmentalization of HDL functions. This is one of the intriguing questions that present studies are addressing.

## HDL Functionality

As mentioned above, HDL-C measurement does not reflect its functionality. The complex composition leads to several HDL functions which can be measured by bioassay. Here, are some examples of analysis that could be considered for the measurement of HDL functionality.

### HDL and Reverse Cholesterol Transport (RCT)

The original pathway delineated by Glomset (1968) involves the physiological removal of cholesterol from peripheral tissues and cells and transportation by HDL to the liver for excretion in the bile and feces. RCT prevents the onset of atherosclerotic plaques and lesions which would result from exaggerated uptake by activated macrophages (Yu J. et al., 2013). Macrophage-specific RCT to apoAI is the critical step for RCT and is routinely described as being the main conduit for the atheroprotective actions of HDL (Rader et al., 2009). In addition, recent evidence also suggests that the biogenesis of HDL, mediated by ABCA1, also facilitates the release of microparticles, contributing up to 30% of apoAI-driven cholesterol efflux (Hafiane and Genest, 2017).

Cholesterol efflux capacity exhibits a robust, inverse relationship with prevalent coronary and peripheral atherosclerosis across human studies (Yvan-Charvet et al., 2007; Out et al., 2008; Tall et al., 2008; Khera et al., 2011; Ishikawa et al., 2015), as well as with incident atherosclerotic cardiovascular events (Rohatgi et al., 2014; Saleheen et al., 2015). Crucially, the findings of Rohatgi et al. (2014) demonstrate how cholesterol efflux capacity was an independent predictor of incident cardiovascular events, and was maintained following adjustment for HDL-C concentrations (Rohatgi et al., 2014). Measurement of cholesterol efflux capacity is currently the most promising assay to define one aspect of HDL functionality. The impact of lipid lowering drugs on cholesterol efflux capacity was recently summarized by Brownell and Rohatgi (2016).

### HDL and Antioxidant Function

High-density lipoprotein prevents accumulation of oxidized LDL which would reduce the structural integrity and function of the endothelium. HDL inhibits metal ion induced oxidation of LDL and lipid peroxidation (Hessler et al., 1979; Parthasarathy et al., 1990; Navab et al., 2000). A key structural component of HDL associated with its antioxidant activity is PON1 which diminishes lipid peroxide formation (Mackness et al., 1991, 1993). PON1 activity is associated with a decrease in the risk of cardiovascular event (Ayub et al., 1999; Ansell et al., 2003; Mackness et al., 2003). In hypertensive patients, however, a recent study concluded that increased hypertensive risk was independently associated with HDL-C and not with PON1 activity (Kunutsor et al., 2017). However, epidemiological studies continue to demonstrate that polymorphisms influence PON1 activity, the most significant being R192Q genotype. HDL from individuals with 192QQ homozygote are the most effective to inhibit LDL oxidation (Durrington et al., 2001; Wheeler et al., 2004). Measuring PON1 activity in serum is an attractive means to monitor the antioxidant capacity of HDL.

### HDL Anti-inflammatory Function

In addition to a number of antioxidant effects, HDL also serves as a powerful mediator of the cellular inflammatory and anti-thrombotic responses. Activated monocytes release inflammatory factors that can induce endothelial dysfunction, characterized by an increase of adhesion molecules expression, such as vascular cell adhesion molecule (VCAM), intercellular cell adhesion molecule (ICAM), and E-selectin (Calabresi et al., 2002; Barter et al., 2004). The ability of HDL to down-regulate the expression of these adhesion molecules has served to evaluate its anti-inflammatory capacity (Cockerill et al., 1995; Calabresi et al., 2002; Gomaraschi et al., 2008; Woudberg et al., 2016).

### HDL Anti-thrombotic Function

Platelet activating factor (PAF) is a potent activator of platelets, monocytes, and leukocytes (Stafforini et al., 1987). The metabolism of PAF in the blood is almost completely regulated by the enzyme PAF acethyldrolase (PAF-AH), which is a structural component of both LDL and HDL. The measurement of PAF-AH activity associated with HDL may reflect its potential anti-thrombotic activity (Stafforini et al., 1987).

Another primary anti-thrombotic action of HDL is activation of prostacyclin (PGI2) release. PGI2 is an arachidonic acid-derived lipid mediator and is a powerful inhibitor of platelet activation. PGI2 promotes smooth muscle relaxation and reduces the release of growth factors that promote smooth muscle cell proliferation (Viñals et al., 1999). HDL increases PGI2 release by endothelial cells via at least two mechanisms. These involve HDL cholesteryl esters serving as arachidonic acid donors for PGI2 production by the cyclooxygenase-2 (COX-2) enzyme and by increases in COX-2 expression (Fleisher et al., 1982; Vinals et al., 1997; Cockerill et al., 1999; Escudero et al., 2003; Martínez-González et al., 2004). Regarding the mechanism of action, moreover, S1P increases the production of cyclic adenosine monophosphate (cAMP) in smooth muscle cells, which induces PGI2 production by increasing COX-2 expression (Damirin et al., 2005).

In addition, HDL limits vasorelaxation through modulation of endothelial nitric oxide synthase (eNOS). Modulation of eNOS activity by HDL has been demonstrated in both cultured endothelial cell and animal models (Besler et al., 2011). Mechanistically, HDL stimulates eNOS activity through scavenger receptor B1 (SRB1) and S1P receptors 1 and 3 (Yuhanna et al., 2001; Nofer et al., 2004; Igarashi et al., 2007). HDL induces Akt phosphorylation, extracellular signal-regulated kinase (erk)1/2 and intracellular calcium ion release, which play roles in a sequence of activation steps leading to phosphorylation of eNOS at Ser-1177 (Mineo and Shaul, 2003; Nofer et al., 2004).

In summary, several assays on HDL functionality may improve the prognosis of future cardiovascular events (O’Neill et al., 2015a). Although HDL functionality measurement may be an approach to evaluate risk of cardiovascular event, most are bioassays. A wide variety in protocols used for similar assays reduces the reproducibility and their assessment is time-consuming, thus making their present use for obtaining rapid diagnostic value limited. Therefore, the use of subclass determination or the enzyme activity may be more practical and faster to use in diagnostic circumstances.

### Altered HDL Functionality

The concept of dysfunctional HDL relates to a total loss of HDL function where the normal anti-atherogenic lipoprotein starts displaying pro-atherogenic properties, often as a result of structural changes (Kontush et al., 2013; Serban et al., 2014; O’Neill et al., 2015b; Rosenson et al., 2016), reviewed by Lüscher et al. (2014). Dysfunctional HDL was first demonstrated during acute phase response in patients following cardiac surgery (Van Lenten et al., 1995) where HDL had a loss in PON1 and PAF-AH activities, combined with a loss in the apoAI content, rendering it pro-inflammatory (Van Lenten et al., 1995). Similarly, during acute phase response, serum amyloid A (SAA), a pro-inflammatory protein, replaces apoAI in HDL structure (Cabana et al., 1996). Binding of SAA to proteoglycans can immobilize HDL in the arterial wall, preventing it from performing anti-atherogenic and anti-inflammatory functions (Lewis et al., 2004; Han et al., 2016). It has been recently shown that increased SAA content in HDL results in increased CVD risk with SAA modifying vascular properties of HDL (Zewinger et al., 2015). Triglyceride enrichment in the HDL core, in patients with CVD and during acute phase response can also cause inhibition of HDL function (Cabana et al., 1996; Brites et al., 2000). Triglyceride content may alter apoAI conformation, limiting access of the central and C-terminal regions to the surface, causing inhibition of apoAI and consequently, HDL functions (Curtiss et al., 2000).

Subsequent to findings from acute phase response, a number of other pathologies and conditions have elucidated the phenomenon of dysfunctional HDL. Dysfunctional HDL is characteristic in patients with CAD, presenting pro-inflammatory HDL phenotype when compared to controls (Ansell et al., 2003; Besler et al., 2011; Holy et al., 2014; Sattler et al., 2015). In chronic renal disease, the HDL functions are impaired and capacity to promote cholesterol efflux, the antioxidant and anti-inflammatory effects are diminished compared to HDL from control subject (Vaziri, 2015). Recent data suggested the role of carbamylation in this process (Sun et al., 2016). We and others have demonstrated that HDL can become dysfunctional in patients with diabetes mellitus as a result of structural changes including HDL glycation (Nobécourt et al., 2010; Brinck et al., 2016) and a truncated form of apoAI (Cubedo et al., 2015; Estruch et al., 2017). This has also been shown in patients with insulin resistance whilst smoking has been linked with producing dysfunctional HDL subclasses with increased susceptibility to glycation (McMillen et al., 2005; Song et al., 2015). Additionally platelets can modify native HDL, resulting in a dysfunctional and pro-thrombotic form (Blache et al., 2012). Patients with familiar hypercholesterolemia display reduced concentrations of apoAIV and LCAT and a truncated form of apoLI (Cubedo et al., 2016; Badimon et al., 2017). Experimental studies showed that much of the structural modifications and impairment in HDL function are as a result of increased LDL cholesterol (LDL-C) concentrations (Vilahur et al., 2015; Padró et al., 2017). HDL dysfunction is a new aspect of HDL metabolism reflecting its complexity and requires further investigation to analyze the effects of disease on HDL function.

## Which HDL Subclass To Improve Protective Capacity?

Allocating activities to discrete HDL particles appears as an attractive means of compartmentalizing the functional diversity of HDL. It may also refine our understanding of the association of HDL with cardiovascular risk if one considers that particle specific changes, rather than global changes in HDL, are linked to risk. In order to address this point, we will compare below the functional effectiveness and risk prediction capacity of the cholesterol content and relative levels of HDL2 and HDL3.

## HDL2 and HDL3 Subclasses

Early epidemiological studies describe HDL2 as more accurate risk indicator for CVD. Indeed, myocardial infarction survivors had significant decreases in HDL2 (Brugger et al., 1986), and HDL2 was inversely correlated with coronary heart disease risk (Johansson et al., 1991). A large study of 4594 healthy patients demonstrated that a decrease in HDL2 was associated with increased CVD risk (Musunuru et al., 2009) and patients with acute coronary syndrome displayed decreased levels of HDL2 and increased levels of HDL3 (Tian et al., 2014). These modern studies continue to argue for HDL2 as a risk factor, although discrepancies continue to confound the argument.

In contrast, *post hoc* analysis of two prospective studies, the IDEAL (Incremental Decrease in End Points through Aggressive Lipid Lowering) trial and the EPIC (European Prospective Investigation into Cancer and Nutrition)-Norfolk case-control study, showed a very high concentration of HDL2 particles, when not accompanied by a correspondingly high level of apoAI containing HDL (i.e., over-enrichment of HDL in cholesterol), may be associated with increased rather than decreased cardiovascular risk (van der Steeg et al., 2008). Kavo et al. (2012) studied HDL from patients who survived a myocardial infarction (MI) at a young age (≤35 years) and healthy control subjects and showed that MI patients had reduced preβ-1 and HDL3 and elevated HDL2 (Kavo et al., 2012). Martin et al. (2014) analyzed the data from two cohorts, the Translational Research Investigating Underlying disparities in acute Myocardial infarction Patient’s Health Status (TRIUMPH) and Intermountain Heart Collaborative Study (IHCS), which indicated that HDL3, rather than HDL2 and total cholesterol, was an improved negative predictor of mortality in myocardial infarction patients (Martin et al., 2014). These data are confirmed by the recent results of the secondary analysis of the AIM-HIGH Study which indicate that the levels of HDL3 and no other lipoprotein fractions are predictive of cardiovascular events (Albers et al., 2016). Further, Ditah et al. (2016), showed that smaller HDL particles, quantified by nuclear magnetic resonance, are inversely independently associated with coronary artery calcification and represents a protective subpopulation (Ditah et al., 2016).

It is clear that controversies exist between preclinical and clinical data regarding the beneficial influences of HDL2 and HDL3. However, when considering the biochemical basis for improved protection, it becomes clear that HDL3 may be the better candidate.

The majority of preclinical studies demonstrate stronger beneficial effects of HDL3 compared to HDL2. Early animal studies indicated that *in vivo* administration of smaller HDL subclasses inhibited the development of atherosclerotic lesions in cholesterol-fed rabbits (Badimon et al., 1989, 1990). Further, an *in vitro* study, HDL3 inhibited LDL oxidation better than HDL2 (Kontush et al., 2003). Shuhei et al. (2010), evaluated *in vitro* the kinetics of copper sulfate-induced oxidation of HDL subclasses in human subjects. HDL3 subclass was less prone to oxidation than HDL2. This may be explained by a higher PON1 activity observed in HDL3 (Shuhei et al., 2010). HDL3 also inhibited tumor necrosis factor alpha (TNF-α) – induced inflammation more effectively than HDL2 (Ashby et al., 1998). HDL3 was functionally superior to HDL2 in all functionality assays including cholesterol efflux capacity, antioxidant, anti-thrombotic, and anti-apoptotic properties (Camont et al., 2013). The results of this study provide the strongest evidence for HDL3 being the functionally superior HDL subclass.

An explanation of the more beneficial effects of HDL3 can be found in its composition (see **Table 1**). Among the components of HDL3, PON1, apoJ, and S1P that have been shown to be protective, while HDL2 contains apoCIII which has been associated with a higher risk of cardiovascular events (Riwanto et al., 2013). In the next section, we will review evidence that raising-HDL drugs should focus more on increasing HDL3 subpopulation than total HDL-cholesterol.

## Selective HDL Subclass May Explain Discrepancies in Therapies Targeting An Increase in HDL

### Statins

Statins inhibit the hepatic synthesis of cholesterol through inhibition of 3-hydroxyl-3-methyl-glutaryl-coenzyme A (HMG CoA) reductase (Istvan and Deisenhofer, 2001). Statins have the most widespread application of the different lipid lowering agents owing to an active reduction in LDL-C levels (Gotto and Opie, 2005). While the major beneficial effect of statins is attributed to its strong capacity to decrease LDL-C levels, a secondary action shows a small increase (approximately 5–10%) in HDL-C levels, however, in patients with low HDL-C levels statins treatment did not seem to improve CVD risk (Jafri et al., 2010). Indeed, in patients treated with statins, non-HDL-C, and apoB concentrations were improved predictors of future major cardiovascular events (Boekholdt et al., 2012). In addition, minor increases in HDL-C in statins-treated patients seems to specifically relate to an increase in HDL2 rather than HDL3. The results of these studies on HDL plasma levels are summarized in **Table 2**.

**Table 2:** Impact of statin and CETP inhibitor therapy on patient lipid profile.

Drug	Disease	LDL-C (%)	HDL-C (%)	HDL2/large HDL (%)	HDL3/small HDL (%)	Reference
Simvastatin	None	–40	Null	+18	Null	Berthold et al., 2014
	Hypercholesterolemia	–49	+6	+28	–12	Neuman et al., 1991
	Hypercholesterolemia	–39	Null	+61	Null	Gaw et al., 1993
	Hypercholesterolemia	–31	+7	+30	+12	Johansson et al., 1991
	High risk CVD	–38	+6	Null	Null	Franceschini et al., 2007
	Familiar Hyperlipoproteinemia	–33 (LDL1)/-23 (LDL2)	+6	+10	Null	Homma et al., 1995
Atorvastatin	Type 2 diabetic patients with ischemic heart disease	–47	+16	+39	–10	Soedamah-Muthu et al., 2003
Pravastatin	Familiar hyperlipidemia	–32	+6	+73	–8	Franceschini et al., 1994
	Familiar hyperlipidemia	–36	Null	Increase in HDL2:HDL3 ratio		Guérin et al., 1995
	Hypercholesterolemia	–18	Null	–10	+6	Cheung et al., 1993
Torcetrapib	Familiar hypercholesterolemia	–14	+54	+157	+46	Kastelein et al., 2007
Anacetrapib	None	–26	+82	+373 (HDL2b)	+15	Krauss et al., 2012

*Statins and CETP inhibitors therapies used to treat cardiovascular disease are summarized regarding their effects on LDL-C and HDL-C as well as HDL subclass specific changes.*

### Cholesterylester Transfer Protein (CETP) Inhibitors

Some of the most promising HDL-raising drugs are the CETP inhibitors. CETP is a hydrophobic glycoprotein mainly secreted from the liver and circulating in plasma mainly bound to HDL. CETP reduce circulating HDL-C levels by transferring cholesteryl ester from HDL to larger lipoproteins, such as chylomicrons, very low density lipoprotein (VLDL) and LDL, in exchange for triglyceride. Four CETP inhibitors have reached late-stage clinical development: torcetrapib, dalcetrapib, anacetrapib and evacetrapib, mostly with disappointing results. Indeed meta-analysis concluded that current CETP inhibitors did not reduce cardiovascular mortality (Verdoia et al., 2015). Most recently, the Randomized Evaluation of the Effects of Anacetrapib through Lipid Modification (REVEAL) trial provided promising results (HPS3/TIMI55–Reveal Collaborative Group Bowman et al., 2017). The REVEAL trial found that the primary outcome (first major coronary event) occurred significantly less frequently in patients with atherosclerotic vascular disease treated with anacetrapib after 4.1 years of follow up (HPS3/TIMI55–Reveal Collaborative Group Bowman et al., 2017). This study showed a 104% increase in HDL-C and a reduction of 17 mg/dl of non-HDL-C in the anacetrapib group compared to the placebo group. In the conclusion, the authors argued that the reduction in non-HDL-C would be anticipated due to relative reduction in the risk of coronary death or myocardial infarction which was observed. This result reduces the likelihood that other actions of anacetrapib played a major role in modifying the risk of coronary events. In particular, the higher mean level of HDL-C in the anacetrapib group does not appear to have had as large an effect on coronary events (HPS3/TIMI55–Reveal Collaborative Group Bowman et al., 2017). Despite this, the REVEAL trial along with the majority of CETP inhibitor studies did not examine the effects of CETP inhibitors on HDL subclass. Those trials that did so are summarized in **Table 2**. All indicate that HDL2 is preferentially raised. In addition to this HDL2 increase, evacetrapib monotherapy also increases preβ-1 HDL but to a lesser extent (Nicholls et al., 2015). In a recent study in mice, anacetrapib and evacetrapib were analyzed for their influence on HDL function, HDL subclass distribution and endothelial function. Expectantly, treatment with both drugs raised HDL-C levels while only evacetrapib increased PON1 activity and RCT (Simic et al., 2017). Similarly to human studies, treatment with both drugs showed increases in large HDL subclasses quantified by nuclear magnetic resonance spectroscopy (Simic et al., 2017). The results of the REVEAL trial are certainly promising, although future studies which examine HDL subclass-specific effects of the treatment will be required to further validate these findings in relation to HDL biochemistry. However, at the time of this review, the manufacturer has made the decision to not pursue its development.

### Niacin

Niacin is the most efficient HDL-C raising drug and mechanisms of action include non-competitive inhibition of hepatocyte microsomal diacylglycerol acyltransferase-2 (DGAT2), an enzyme which catalyzes the final reaction involved in triglyceride synthesis (Ganji et al., 2002) as well as selective inhibition of apoAI uptake without influencing *de novo* synthesis, which raises HDL-C levels (Jin et al., 1997). Widespread application of niacin treatment has been limited by adverse side-effects including flushing in patients. It seems that niacin therapy both on its own and in combination with statins preferentially raise HDL2 whilst simultaneously raising HDL-C and lowering triglyceride, LDL and VLDL cholesterol levels (Carlson, 2005), **Table 3**. It is important to distinguish these outcomes from statin-related effects, as the propensity of niacin to raise HDL2 was shown to be superior to atorvastatin (Toth et al., 2012).

**Table 3:** Impact of niacin and fibrate therapy on patient lipid profile.

Drug	Disease	LDL-C (%)	HDL-C (%)	HDL2/large HDL (%)	HDL3/small HDL (%)	Reference
Niacin with statin and ezetimbe	CVD	–13	+11	Null	Null	Boden et al., 2011
Niacin and laropiprant with simvastatin	Primary hypercholesterolemia or mixed hyperlipidemia	–45	+20	+38	+14	Ballantyne et al., 2012
Niacin	Dyslipidemia	–35	+15	+82	–4	McKenney et al., 2001
	Primary hypercholesterolemia	–16	+23	+84	Null	Morgan et al., 2003
	Hyperlipidemia	–	–	+102	–2	Toth et al., 2012
Niacin and gemfibrozil	Hyperlipidemia	–20	+32	+90	Null	Superko et al., 2009
Bezafibrate	Coronary artery disease and dyslipoproteinemia	Null	Null	Null	+7	Ruotolo et al., 1998
Ciprofibrate	Hyperlipoproteinemia	–17	+13	Null	+22	Guérin et al., 2003
Fenofibrate	No diabetic patients	Null	+22	–2.3	+1.9	Franceschini et al., 2007

*Niacin and fibrate therapies used to treat cardiovascular disease are summarized regarding their effects on LDL-C and HDL-C as well as HDL subclass specific changes.*

### Fibrates

Fibrates do not reduce LDL-C to the same extent as statins, however, they are still widely prescribed, in many cases as a secondary treatment in combination with statins (Gotto and Opie, 2005; Moutzouri et al., 2010; Katsiki et al., 2013). The mechanism of the multiple actions of fibrate activity can be summarized as: induction of lipoprotein lipolysis; induction of hepatic fatty acid uptake; increased removal of LDL particles; inhibition of cholesterol and triglyceride exchange between HDL and VLDL; stimulation of HDL production via induction of hepatic synthesis of apoAI and apoAII and reduced production of VLDL due to reduction of free fatty acid to the liver (Vu-Dac et al., 1995; Berthou et al., 1996; Staels et al., 1998). Conversely to the aforementioned drugs, fibrate treatment may promote increases in HDL3. Unfortunately, these increases in HDL3 are relatively low and may be insufficient to reduce the risk of cardiovascular event. The effect of fibrate treatment on HDL subclass distribution is summarized in **Table 3**.

In summary, traditional lipid-lowering drugs have varied influences on HDL subclass distribution. Whilst it is well-understood that statins, CETP inhibitors, fibrates and niacin raise HDL-C whilst decreasing LDL-C, each cause selective increases in HDL2 or HDL3. Statins, CETP inhibitors and niacin raise HDL2 whilst only the fibrates, in a limited number of studies and to a limited extent, have been shown to increase HDL3. These differences may potentially explain why the clinical trials aimed at attenuating low HDL-C levels have been met with such disappointing results. In most cases, the functionally beneficial HDL3 is not raised in combination with total HDL-C.

As reviewed recently by Muthuramu et al. (2017), none of the aforementioned trials have hard clinical outcomes specifically related to HDL-C. This implies that much of the current pharmaceutical-based therapies have causal effects on HDL-C and are not HDL-targeted. This is in agreement with our argument which postulates that current pharmaceutical interventions are not sufficiently specific to HDL, in particular to raising HDL3 and improving HDL function. In this regard, there is a risk of false negative conclusions about the clinical efficiency of surrogate endpoints and biomarkers which do not sufficiently mimic clinically meaningful endpoints (Muthuramu et al., 2017). Of the current available therapies, we propose how reconstituted HDL (rHDL) therapy serves as a potential novel therapy which can target specific HDL subclasses, thereby improving function and reducing risk. Trials examining rHDL therapies are more specific and have better defined clinical endpoints.

## HDL Raising Therapies and Humoral Autoimmunity Against apoAi, An Unexplored Link?

A growing body of evidence indicates that IgG autoantibodies against apoAI (anti-apoAI IgG) exist in a substantial proportion of the general population (up to 20%) where they represent an independent CVD risk factor (Antiochos et al., 2016) associated with a decreased survival (Antiochos et al., 2017). In different high CVD risk populations associated with or without autoimmune diseases, high levels of anti-apoAI IgG were shown to be independent predictors of major cardiovascular events (Vuilleumier et al., 2010a,b, 2013; Keller et al., 2012; El-Lebedy et al., 2016).

In this context mechanistic studies demonstrated that these antibodies could act as mediators of inflammation, atherogenesis, heart rate dysregulation, and myocardial necrosis through toll-like receptor-2 and CD14 complex signaling (Montecucco et al., 2011, 2015; Pagano et al., 2012, 2016; Mannic et al., 2015), indicating that these antibodies could represent a new CVD therapeutic target amenable to apoAI mimetic peptide-based immunomodulation (Pagano et al., 2015). However, the reason why these autoantibodies could appear in non-autoimmune conditions remained unclear until the publication of a genome-wide association study that highlighted a Fc receptor like (FCRL) 3 single nucleotide polymorphism as a key genetic determinant underlying the existence of anti-apoAI IgG in the general population (Antiochos et al., 2017). FCRL3 being a major autoimmune susceptibility gene in human, this study provided the first biological rational to explain the existence of these antibodies in humans. Concomitantly, a small-sized phase-two randomized-controlled trial (EXLPORE) set the proof of principle that HDL-raising therapies (niacin in this case), could induce the production of a sustained and specific anti-apoAI IgG response associated with a loss of the antioxidant function of HDL (Batuca et al., 2017). Given the fact that most, if not all, HDL-raising therapies induce important conformational/size changes of HDL, humoral autoimmune response to apoAI may well-represent a generic effect of most HDL-raising therapies. Interestingly, the EXPLORE trial showed that niacin did not affect anti-HDL antibodies suggesting that the structural changes of the major protective fraction of HDL (apoAI) may drive this autoimmune response, rather than a change in HDL size.

Given the strength of associations reported between anti-apoAI IgG, CVD, and HDL dysfunction, it is tempting to speculate that in genetically prone individuals the humoral autoimmune response induced by HDL-raising therapies could jeopardize the efficacy of such therapeutic modality. Addressing this point in a systematic manner in future HDL-related studies would certainly be welcome given the current paucity of data on this under-explored topic.

## Novel Therapy to Target Protective HDL Subclass

Since it can be postulated that particle specific changes are more closely related to cardiovascular risk, novel therapies addressing this are in development which may accomplish improved protection. In this section, we hypothesize an example of such a therapy which may allow for improved cardioprotection owing to a selective increase in cardioprotective HDL subclasses. In this context, In this context, rHDL may be an option. Originally, rHDL composed of apoAI and phospholipids (Jonas, 1985), was exploited for years in experimental laboratory settings to investigate HDL function. It presents an attractive model to test the roles of individual peptide and lipid components of HDL. Examples of current rHDL therapies include CER-001 (an HDL mimetic agent), CSL112 (an infusible, plasma derived apoAI) and ACP-501 (recombinant human LCAT). These agents have been separately tested and have shown good tolerability with no adverse side effects in patients (Gibson et al., 2016; Shamburek et al., 2016; Keyserling et al., 2017). In patients, administration of rHDL was associated with reduction in plaque size, better endothelial function and increase in anti-inflammatory markers (Nissen et al., 2003; Tardif et al., 2007; Nieuwdorp et al., 2008; Shaw et al., 2008; Patel et al., 2009). rHDL were used in patients with acute coronary syndrome and lead to an increase in plasma HDL-C and a decrease in a decrease in LDL-C (Chenevard et al., 2012). As a caveat to these positive findings, animal studies and HDL-targeted gene therapy studies have indicated that apoAI overexpression did not cause regression of pre-existing atherosclerotic lesions but rather retarded further expansion of pre-existing lesions (Rong et al., 2001; Li et al., 2011; Van Craeyveld et al., 2011). Additionally, a study analyzing CER-001 found no difference between placebo and treatment in the reduction of atheroma volume (Tardif et al., 2014). The effects of CER-001, may however, be dose-dependent (Keyserling et al., 2017). Importantly, addition of rHDL improved HDL RCT in a number of studies (Gibson et al., 2016; Zheng et al., 2016; Keyserling et al., 2017). The composition of rHDL resembles the preβ-1 HDL particle and is highly modifiable. rHDL can absorb many products including cholesterol, proteins, and S1P *in vivo*. These observations strongly suggest that rHDL can therefore be protective through improving RCT or by absorbing beneficial proteins.

It would be interesting to assess the ability to improve these beneficial capacities by modulating rHDL composition. In this context our group has investigated the beneficial effect of the addition of S1P to basal rHDL. We demonstrated experimentally that adding S1P improves the cardiac survival capacity *in vitro, ex vivo*, and *in vivo* (Frias et al., 2010; Brulhart-Meynet et al., 2015). The HDL3 subclass, contains 2–3 times more S1P than HDL2 (Kontush et al., 2007; Lee et al., 2010). It seems that the S1P content influences HDL-induced cardioprotection and that S1P-enriched rHDL offer a better protection (Brulhart-Meynet et al., 2015). But until now, human studies using rHDL have not considered the S1P content in their preparations but rather focused on the apoAI content. In addition, the peptide components of HDL outlined in this review (such as PON1, apoJ, or apoM) could be added to better improve cardioprotective capacities. In this regard, engineering a functionally superior rHDL may be possible for patient treatment. This intervention has been shown to improve cardioprotection in patients (see review, Darabi et al., 2016). Some studies have also indicated that infusion with rHDL can influence apoAI concentrations and lipidome profiles of native HDL (Nanjee et al., 1999); increase the concentrations of preβ-1 HDL (Nanjee et al., 2001) and improve anti-inflammatory function of native HDL (Patel et al., 2009). More detailed information on the apoAI-directed therapies can be found in the review (Millar and Cuchel, 2015).

## Conclusion

**FIGURE 2:**
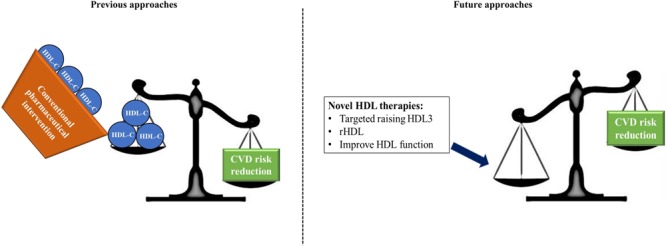
Summary of our current findings. Previous approaches which aimed to improve CVD risk focussed on pharmaceutical increases of HDL-C concentrations. These approaches have largely been met with failure due in part to the inherent complexity of the HDL particle which displays diverse function and heterogeneity. We propose that future approaches should focus on targeted increases in HDL3, which we suggest as the functionally superior HDL subclass; using reconstituted HDL, containing increased concentrations of protective apoAI and S1P; and overall a focus on improvement of HDL function without necessarily raising HDL-C.

We have discussed in this review that HDL is an extremely complex particle composed of an array of lipids and peptides which result in functionally and structurally distinct HDL subclasses. We have suggested, similar to Kontush and Chapman (2006), that the smaller HDL subclass, HDL3 performs a functionally superior role to the larger HDL2 owing in particular to increased association with cardioprotective enzymes and lipids such as S1P. Drug therapies which raise HDL-C have been met with disappointing results. We recommend that future research focuses to a greater extent on HDL functionality and subclass distribution. Novel therapies such as rHDL infusion may then permit selectively raising the levels of functionally superior HDL subclasses thereby reducing cardiovascular risk in patients (**Figure 2**).

## Author Contributions

NW assimilated and compiled the final manuscript which was written in part by SP, SL, RS, NV, RJ, and MF. All authors contributed equally in review of the final manuscript.

## Conflict of Interest Statement

RS reports personal fees from Amgen, personal fees from Recordati, grants and personal fees from Sanofi, outside the submitted work. The other authors declare that the research was conducted in the absence of any commercial or financial relationships that could be construed as a potential conflict of interest.

## Abbreviations

ABCA1 and ABCG1: ATP binding cassette A1 and G1
ApoAI: apolipoprotein AI
CAD: coronary artery disease
CETP: cholesterylester transfer protein
COX-2: cyclooxygenase-2
CVD: cardiovascular disease
HbA1c: glycated hemoglobin
HDL-C: HDL cholesterol
HMG CoA: 3-hydroxyl-3-methyl-glutaryl-coenzyme A
ICAM: intercellular cell adhesion molecule
LCAT: lecithin-cholesterol acyltransferase
LDL-C: LDL cholesterol
PAF: platelet activating factor
PAF-AH: platelet activating factor acetylhydrolase
PGI2: prostacyclin
PON1: paraoxonase
RCT: reverse cholesterol transport
rHDL: reconstituted HDL
S1P: sphingosine-1-phosphate
SAA: serum amyloid A
VCAM: vascular cell adhesion molecule
